# Metastasis of Hepatocellular Carcinoma to the Esophagus: Case Report and Review

**DOI:** 10.1155/2018/8685371

**Published:** 2018-09-18

**Authors:** Jun-ichiro Harada, Takeshi Matsutani, Nobutoshi Hagiwara, Yoichi Kawano, Akihisa Matsuda, Nobuhiko Taniai, Tsutomu Nomura, Eiji Uchida

**Affiliations:** Department of Gastrointestinal and Hepato-Biliary-Pancreatic Surgery, Nippon Medical School, 1-1-5 Sendagi, Bunkyo-ku, Tokyo 113-8603, Japan

## Abstract

A follow-up endoscopy in a 71-year-old Japanese man who had undergone a left lateral segmentectomy for HCC two years ago revealed an approximately 2 cm in diameter pedunculated polypoid mass in the middle part of the thoracic esophagus. Immunohistochemical staining of the endoscopic biopsy revealed a metastatic HCC esophageal tumor. As the patient's disease could be radically removed by preoperative examinations, we resected the metastatic esophageal tumor via right thoracotomy and esophagogastrostomy reconstruction. Histological examination of the resected specimen revealed that the esophageal tumor was compatible with a HCC metastasis. This is an extremely rare case of a solitary metastasis to the esophagus from HCC in the literature.

## 1. Introduction

Extrahepatic metastasis of hepatocellular carcinoma (HCC) is relatively rare, even in advanced HCC cases that have intrahepatic metastases. The lung, bone, and adrenal gland are the most common sites of distant HCC metastases via hematogenous metastasis through the hepatic artery or portal vessel system [1–3]. The incidence of gastrointestinal tract metastases from HCC is low, with several groups reporting rates between 0.5% and 2%; however, these patients usually have poor outcomes [2, 4, 5]. HCC metastasis to the gastrointestinal tract most commonly involves the duodenum, followed by the stomach and the colon. Esophageal HCC metastases are extremely rare; the reported rates in the literature are less than 0.4% [1–3, 6]. The clinicopathological characteristics of HCC with esophageal involvement remain unknown. Herein, we present a surgically treated case of esophageal metastasis from HCC.

## 2. Case Report

**Figure 1:**
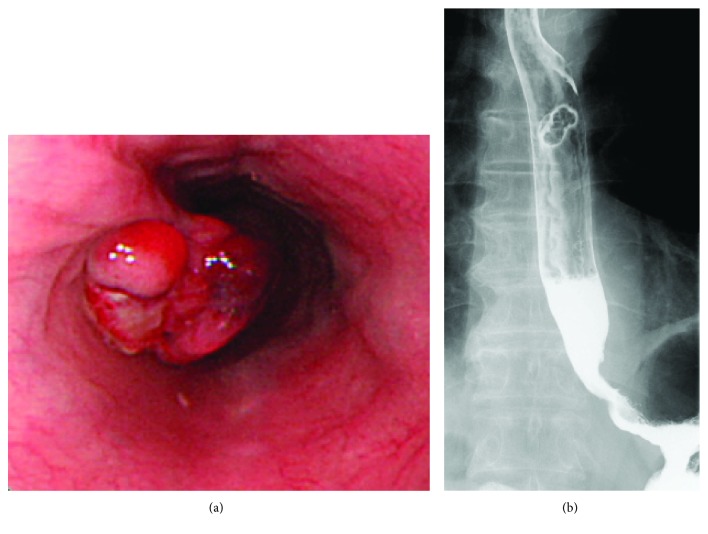
(a) Upper gastrointestinal endoscopy showing a pedunculated polypoid tumor in the middle thoracic esophagus. (b) Barium esophagography revealing an elevated lesion in the middle thoracic esophagus.

**Figure 2:**
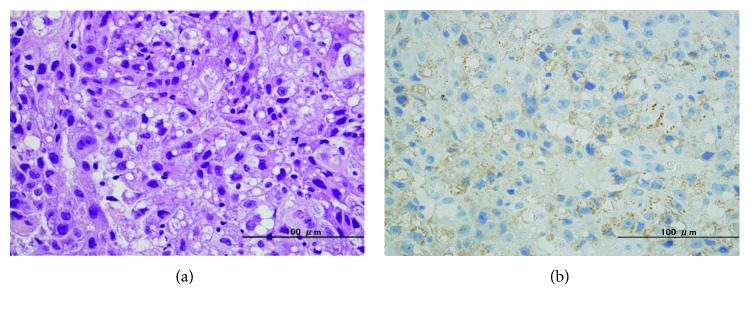
(a) Pathological findings of biopsy specimens; tumor cells with acidophilic cytoplasm proliferated without a tubular structure. (b) Tumor cells showing immunopositive for hepatocyte stain.

**Figure 3:**
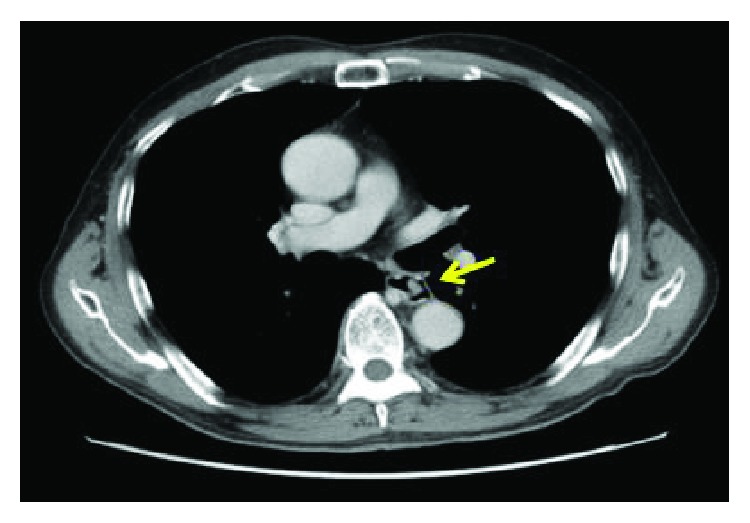
Chest computed tomography showing an elevated mass in the esophageal lumen (arrow).

**Figure 4:**
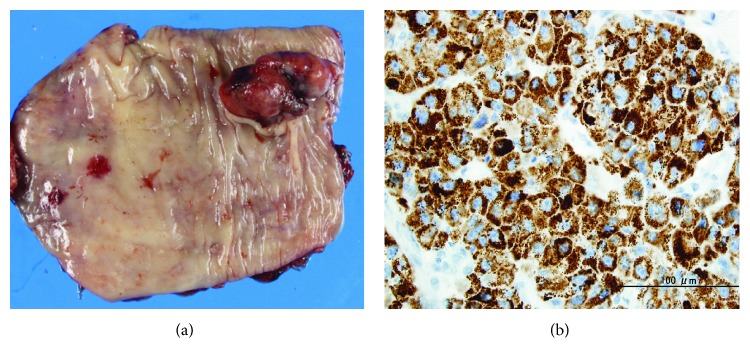
(a) By gross appearance, the resected specimen shows a reddish polypoid tumor in the middle esophagus. (b) Metastatic hepatocellular carcinoma to the esophagus confirming by positive AFP staining.

A 71-year-old Japanese man with a medical history of HCC that resulted from chronic hepatitis B infection underwent a left lateral segmentectomy for HCC at another institute. Pathological findings of the resected specimens were moderately differentiated hepatocellular carcinoma (St-P, 55 × 50 × 38 mm, eg, fc(+), fc-inf(+), sf(−), s0, nx, vp1, vv0, va1, b0, im0, p0, sm(−), and lc lead to pT3 and pStageIII). Two years after surgery, his serum alpha-fetoprotein (AFP) level increased to 1800 ng/ml (normal is 0–10 ng/ml). Physical examination showed no remarkable abnormal findings. Laboratory blood and chemical examination results were also within normal limits. A follow-up examination that included an upper gastrointestinal endoscopy showed a pedunculated polypoid tumor in the middle thoracic esophagus, approximately 2 cm in diameter (Figure 1(a)). Esophageal varices were not seen at the anal side of the tumor. A barium esophagogram showed an elevated mass in the middle thoracic esophagus (Figure 1(b)). The biopsy specimen obtained from the esophageal lesion revealed tumor cells with acidophilic cytoplasm that proliferated without a tubular structure (Figure 2(a)). Tumor cells in the biopsy specimens were positive for hepatocyte stain (monoclonal mouse anti-human hepatocyte antibody) (Figure 2(b)). The esophageal tumor was diagnosed as a metastatic HCC tumor. Chest computed tomography (CT) showed an elevated mass in the esophageal lumen (Figure 3). Abdominal CT detected no evidence of metastasis to the lung or of new HCC lesions in the liver, except for lymph node metastases in the lesser curvature area of the stomach. However, a portal tumor thrombus was not found. As the patient was in good general condition and preoperative imaging showed resectable disease, we performed surgical resection. Esophageal resection via right thoracotomy was performed with regional lymph node dissection, and the whole stomach for reconstruction was made to provide better protection of the submucosal vessels, compared to gastric tube approach. Esophagogastrostomy was performed at the intrathorax where the gastric tube was lifted up through the posterior mediastinal route. Intraoperative exploration revealed no peritoneal dissemination. The gross appearance of the resected specimen was a reddish polypoid tumor in the middle esophagus (Figure 4(a)). A metastatic esophageal tumor from HCC was confirmed by positive immunohistochemical staining for hepatocyte and AFP (Figure 4(b)). Two months following the operation, a follow-up CT demonstrated multinodular-type HCC in both lobes of the liver. The patient received no additional therapies and died from disease progression two months following the operation.

## 3. Discussion

Metastatic esophageal tumors are extremely rare in living patients with malignant tumors. A study of 1835 autopsy cases from a variety of malignancies revealed 112 (6.1%) esophageal metastases, and the most common malignancies to produce such metastases were the lung (41/450, 11.3%), breast (14/188, 7.4%), and ovarian cancer (1/40, 2.5%) [7]. In contrast, HCC frequently invades the vascular spaces of the liver; consequently, extrahepatic HCC metastases are rare. The most common extrahepatic HCC metastases diagnosed in autopsy or surgical series were to the lung (18.1%–49.2%) followed by lymph nodes (26.5%–41.7%), bone (4.2%–16.3%), and adrenal glands (8.4%–15.4%) [1, 3, 6]. Incidences of gastrointestinal metastases are infrequent, and metastatic esophageal tumors from HCC are extremely rare, present in less than 0.4% of HCC patients [1–3, 6]. Despite improvements in diagnostic techniques, only 13 cases of esophageal metastasis in living HCC patients have been reported in the last 20 years on PubMed, including this case (Table 1) [8–18].

The major symptoms of esophageal HCC metastases were gastrointestinal bleeding and dysphagia. Upper gastrointestinal endoscopy is necessary to diagnose esophageal HCC metastases because three previously reported cases, including ours, were asymptomatic [11, 16]. Metastatic esophageal tumors are usually located in the submucosal layer [7]; therefore, esophagography and endoscopy show severe luminal stricture with normal overlying mucosa, which often complicates histological diagnoses. A literature review indicated that the most common endoscopic findings of esophageal HCC metastases were polypoid or submucosal tumors [9, 14, 15]. If an endoscopic biopsy reveals a lack of available tumor cells, several imaging modalities, such as CT, endoscopic ultrasound, or angiography, are necessary to differentiate between primary esophageal cancer and HCC metastases [9]. In this case, the endoscopic and imaging findings were compatible with a metastatic esophageal tumor of HCC origin.

The exact mechanisms involved in esophageal metastasis from HCC are still unknown; however, several possibilities have been proposed. The main possible routes for esophageal involvement are thought to be a direct extension from metastatic mediastinal nodes or spread in the esophageal wall through systemic hematogenous pathways in HCC. The spread of HCC is characterized by disseminating tumor infiltration and the frequent occurrence of tumor thrombi in the portal vein [19]. Moreover, multinodular-type HCC frequently involves portal vein invasion and intrahepatic metastasis [2]. Esophageal metastasis from HCC is presumed to be caused by tumor thrombi invading through the portal system and dissemination from the hepatofugal portal blood flow to the gastrointestinal tract [4, 19]. As HCC frequently invades the portal vein, the involvement of the gastrointestinal tract, including the esophagus, by metastatic HCC via the portal system may not be uncommon. Arakawa et al. [19] found 12 cases (38.7%) that had variceal tumor thrombi among 55 HCC autopsy cases. Thus, esophageal metastasis that is caused by tumor thrombi infiltrating via the portal system and disseminating by hepatofugal portal blood flow might be a route of hematogenous spread. In this case, histological examination of resected specimens did not demonstrate any specific features directly related to these mechanisms, and no existence of portal thrombus was detected on the presurgical abdominal CT scan. Thus, a causative mechanism involved in metastasis to the esophagus from HCC in our case cannot be made at this time. We speculated that this case involved spreading by hepatofugal blood flow because there was no evidence of direct invasion or lymphadenopathy around the esophagus.

Advanced hepatocellular carcinoma (HCC) is one of the most deadly diseases with few systemic therapeutic options. Sorafenib is the first-line molecular target treatment of patients with advanced HCC and increases overall survival by approximately 3 months (10.7 months) compared with placebo (7.7 months). The RESORCE trial demonstrated that regorafenib in the second-line treatment increased from 7.8 months with placebo to 10.6 months with regorafenib after patients experienced disease progression on sorafenib. However, other newer molecular agents have failed to demonstrate significantly improved long survival in clinical trials. The effective systemic, immune, or etiology-specific therapies of patients with advanced HCC have not been established. Despite improvements in therapeutic techniques, a standard treatment for esophageal HCC metastases has not been also established. As the majority of these patients already have terminal disease with distant metastases at multiple sites, palliative chemotherapy or radiation therapy is usually the first treatment choice. The interval between diagnosis of esophageal metastasis and death was short in the 13 reviewed cases, with a mean of only 5.5 months. Thus, esophageal metastasis of HCC has an extremely poor prognosis, and therapy for these HCC cases should be individualized and tailored, whereas HCC is common in Asia. Metastatic HCC generally shows poor responses to chemotherapy and radiation. Several reports have shown that tumor resection can provide excellent palliation and long-term survival in certain cases without metastases to other sites [7, 8]. The resection of metastatic tumors might prolong survival in patients with esophageal metastases from HCC who can tolerate aggressive treatments. If our patient had no lymph node metastases, we could perform an endoscopic submucosal dissection that gives less surgical stress. Our patient had rapid disease progression by two months postsurgery, and it should be discussed with patients whether surgical removal is the optimal treatment.

## 4. Conclusion

We report a rare case of a preoperatively diagnosed esophageal metastasis from HCC. Specialists in digestive organ diseases should be aware that esophageal metastasis of HCC may exhibit the aforementioned endoscopic characteristics and may cause dysphagia or gastrointestinal bleeding, although this case showed no symptoms from the metastatic esophageal tumor.

## Figures and Tables

**Table 1:** English literature review of esophageal hepatocellular carcinoma metastases.

No.	Author	Year	Age	Sex	Symptoms	Gross type of esophageal tumor	Type of hepatitis	Serum AFP (ng/ml)	Therapy of varices	Therapy for HCC	Therapy for esophageal metastasis	Other metastasis at living	Outcome	Survival time (M)
1	Sohara	2000	54	M	Melena	Submucosal	HCV	4987	Esophageal transection, splenectomy	TAI	−	Lung	Death	3
2			46	M	Hematemesis	Polypoid	NA	990	Esophageal transection, EIS	TACE, radiation	−	−	Death	7
3	Kume	2000	56	M	Dysphagia, tarry stool	Submucosal	HBV	12,200	EIS, EVL	TACE	−	Lung, bone	Death	2
4	Cho	2003	50	M	Dysphagia, hematemesis	Polypoid	NA	Elevated	−	Resection	Radiation, TAI	−	Death	11
5	Tsubouchi	2005	63	M	None	Polypoid	HCV	4130	+	TACE	−	Stomach	Death	7
6	Yan	2007	53	M	Melena	Polypoid	HBV	17,036	−	−	−	−	Death	1
7	Choi	2008	66	M	Melena	Submucosal epolypoid	Non-B, non-C	3.47	EVL	TACE	−	−	Death	7
8	Xie	2008	50	M	Dysphagia, odynophagia	Cauliflower-like	HBV	NA	−	Systemic chemotherapy, OLT, TACE	Radiation	−	Alive	>7
9	Hsu	2009	54	M	Hematemesis, tarry stool	Polypoid	HBV	NA	−	OLT, TACE	−	Stomach	Death	4
10	Kahn	2010	55	M	Dysphagia	Polypoid	HCV	1426	−	OLT, TACE	Hyperthermia, PDT, stent placement	−	Death	10
11	Boonnuch	2011	59	M	Dysphagia	Submucosal	NA	510	−	OLT, TACE	Resection	−	Alive	>2
12	Fukatsu	2012	63	M	None	Polypoid	NA	NA	EVL, EIS	TACE, RFA	−	−	Death	1
13	Our case		71	M	None	Polypoid	HBV	1801	+	Resection	Resection	Lymph node	Death	3

HCV: hepatitis C virus; HBV: hepatitis B virus; NA: not available; EIS: endoscopic injection sclerotherapy; EVL: endoscopic variceal ligation; TAI: transcatheter arterial injection; TACE: transcatheter arterial chemoembolization; OLT: orthotopic liver transplantation; RFA: radiofrequency ablation; PDT: photodynamic therapy.
